# Percent body fat estimations in college women using field and laboratory methods: a three-compartment model approach

**DOI:** 10.1186/1550-2783-4-16

**Published:** 2007-11-07

**Authors:** Jordan R Moon, Holly R Hull, Sarah E Tobkin, Masaru Teramoto, Murat Karabulut, Michael D Roberts, Eric D Ryan, So Jung Kim, Vincent J Dalbo, Ashley A Walter, Abbie T Smith, Joel T Cramer, Jeffrey R Stout

**Affiliations:** 1 Department of Health and Exercise Science, University of Oklahoma, Norman, USA; 2 Department of Pediatrics, University of Oklahoma Health Sciences Center, Oklahoma City, USA

## Abstract

**Background:**

Methods used to estimate percent body fat can be classified as a laboratory or field technique. However, the validity of these methods compared to multiple-compartment models has not been fully established. This investigation sought to determine the validity of field and laboratory methods for estimating percent fat (%fat) in healthy college-age women compared to the Siri three-compartment model (3C).

**Methods:**

Thirty Caucasian women (21.1 ± 1.5 yrs; 164.8 ± 4.7 cm; 61.2 ± 6.8 kg) had their %fat estimated by BIA using the BodyGram™ computer program (BIA-AK) and population-specific equation (BIA-Lohman), NIR (Futrex^® ^6100/XL), a quadratic (SF3JPW) and linear (SF3WB) skinfold equation, air-displacement plethysmography (BP), and hydrostatic weighing (HW).

**Results:**

All methods produced acceptable total error (*TE*) values compared to the 3C model. Both laboratory methods produced similar *TE *values (HW, *TE *= 2.4%fat; BP, *TE *= 2.3%fat) when compared to the 3C model, though a significant constant error (*CE*) was detected for HW (1.5%fat, *p *≤ 0.006). The field methods produced acceptable *TE *values ranging from 1.8 – 3.8 %fat. BIA-AK (*TE *= 1.8%fat) yielded the lowest *TE *among the field methods, while BIA-Lohman (*TE *= 2.1%fat) and NIR (*TE *= 2.7%fat) produced lower *TE *values than both skinfold equations (*TE *> 2.7%fat) compared to the 3C model. Additionally, the SF3JPW %fat estimation equation resulted in a significant *CE *(2.6%fat, *p *≤ 0.007).

**Conclusion:**

Data suggest that the BP and HW are valid laboratory methods when compared to the 3C model to estimate %fat in college-age Caucasian women. When the use of a laboratory method is not feasible, NIR, BIA-AK, BIA-Lohman, SF3JPW, and SF3WB are acceptable field methods to estimate %fat in this population.

## Background

Accurate assessment of body composition is necessary in order to monitor obesity class, nutritional status, training outcomes, and general health [1]. Specifically, fat-free mass and fat mass can be used to identify minimal nutrition requirements and resting energy expenditure [2,3]. Additionally, sports nutrition experts can utilize body composition values to develop specific dietary interventions. Validated laboratory methods, such as hydrostatic weighing (HW), and multiple compartment models, such as the three-compartment (3C) model, are impractical to use in large population studies. Specifically, Wang et al. [4] concluded that the 3C model of Siri [5] was superior to HW using the Brozek [6] equation to estimate percent body fat (%fat) when compared to the six-compartment model, while Fuller et al. [7] determined that the precision between the four-compartment (4C) and 3C model did not differ. However, research is not in total agreement when comparing HW to the multiple-compartment model in adult women [4,8]. Ultimately, both multiple-compartment models and HW entail greater facility requirements and are more costly compared to more convenient field methods, such as skinfolds, near-infrared interactance (NIR), and bioelectrical impedance (BIA). However, research is equivocal or insufficient on the validity of such field methods. In addition, air-displacement plethysmography via the BOD POD^® ^(BP) holds promise as a laboratory method to use in place of HW for populations in which HW is impractical, such as children, the elderly, or those who are ill. However, the validity of the BP compared to the 3C model has not been investigated.

When assessing body composition, NIR and BIA are appealing methods due to the safety, noninvasiveness, and speed of administration when compared to laboratory techniques that require some risk, expensive equipment, and trained personnel [9]. Several studies have examined the validity of NIR [10-15] and BIA [11,12,15-18] with conflicting results. An alternative field method to NIR and BIA is the measurement of skinfold thickness to estimate body density (BD). The popularity of skinfolds has grown, prompting the development of several population-specific, as well as generalized equations. If acceptable agreement is found to exist between established laboratory methods, such as the 3C model, and field methods, such as NIR, BIA, and skinfold equations, these field methods could provide potential alternatives to cumbersome laboratory methods.

The purpose of this study is four-fold: to compare %fat estimations between laboratory methods (BP and HW to the 3C model); to compare a newly-developed device NIR-generated (Futrex 6100/XL) %fat values to the 3C model; to compare the RJL Quantum II (BIA-AK) and the recommended BIA (BIA-Lohman) equation to the 3C model; and to compare commonly used quadratic (SF3JPW) and linear (SF3WB) generalized Sum3 skinfold equations for estimating %fat to the 3C model in college-age Caucasian women.

## Methods

### Participants

Thirty Caucasian women volunteered to participate in the study (Table 1). All body composition measurements were performed on the same day following a 12-hour fast (*ad libitum *water intake was allowed). The subjects were also instructed to refrain from exercising for at least 12 hours prior to testing. Height and weight were measured via a calibrated physician's scale to the nearest 0.5 cm and 0.01 kg, respectively, with the subject wearing a tight-fitting bathing suit. During height measurements, subjects were instructed to extend their toes off the base of the scale and stand as erect as possible to eliminate false height values associated with inconspicuous plantar flexion. Subjects wore the same tight-fitting bathing suit during all body composition measurements. Prior to measuring HW, subjects completed all body composition determinations including BP, BIA, NIR, and SF in no particular order. The purpose of the study and a description of the testing protocol were explained to each subject. Additionally, the study was approved by The Institutional Review Board for Human Subjects, and written informed consent was obtained from each subject prior to testing.

**Table 1:** Description characteristics of the subjects (*n *= 30)

Variable	x¯ MathType@MTEF@5@5@+=feaafiart1ev1aaatCvAUfKttLearuWrP9MDH5MBPbIqV92AaeXatLxBI9gBaebbnrfifHhDYfgasaacPC6xNi=xH8viVGI8Gi=hEeeu0xXdbba9frFj0xb9qqpG0dXdb9aspeI8k8fiI+fsY=rqGqVepae9pg0db9vqaiVgFr0xfr=xfr=xc9adbaqaaeGacaGaaiaabeqaaeqabiWaaaGcbaGafmiEaGNbaebaaaa@2D64@	SD	Range
Age (y)	21.1	1.5	18 – 24
Body weight (kg)	61.2	6.8	46.7 – 77.1
Height (cm)	164.8	4.7	154.0 – 176.0

### Hydrostatic weighing

Body density was assessed from HW with correction for residual volume. Residual volume was determined on land with the subject in a seated position using the oxygen dilution method of Wilmore [19]. Underwater weight was measured to the nearest 0.025 kg in a submersion tank in which a PVC swing seat was suspended from a calibrated Chatillon^® ^15-kg scale (Model # 1315DD-H, Largo, FL.). The average of the 3 highest values from 6 to 10 trials was used as the representative underwater weight. Percent body fat was calculated from BD using the revised formula of Brozek et al. [6] (Table 2).

**Table 2:** Validation of methods of predicting %fat compared to 3C

	Method	% fat (x¯ MathType@MTEF@5@5@+=feaafiart1ev1aaatCvAUfKttLearuWrP9MDH5MBPbIqV92AaeXatLxBI9gBaebbnrfifHhDYfgasaacPC6xNi=xH8viVGI8Gi=hEeeu0xXdbba9frFj0xb9qqpG0dXdb9aspeI8k8fiI+fsY=rqGqVepae9pg0db9vqaiVgFr0xfr=xfr=xc9adbaqaaeGacaGaaiaabeqaaeqabiWaaaGcbaGafmiEaGNbaebaaaa@2D64@ ± SD)	Slope	Intercept	*CE*	*r*	*SEE*	*TE*	Limits
	3C	26.3 ± 4.6							

Lab	HW	24.8 ± 5.6	0.78	6.98	1.5*	0.94	1.6	2.4	5.3, -2.3
	BP	26.7 ± 5.5	0.77	5.91	-0.3	0.91	1.9	2.3	4.1, -4.8

Field	NIR	25.4 ± 3.7	1.12	0.47	0.9	0.82	2.7	2.7	6.1, -4.2
	BIA-AK	26.4 ± 4.4	0.96	0.84	-0.1	0.92	1.9	1.8	3.5, -3.7
	BIA-Lohman	25.4 ± 3.4	1.26	-5.58	0.9	0.93	1.8	2.1	4.7, -2.8
	SF3JPW	23.7 ± 3.9	0.93	4.24	2.6*	0.80	2.8	3.8	8.1, -2.8
	SF3WB	25.5 ± 2.5	1.62	-15.05	0.8	0.88	2.2	2.8	6.0, -4.5

* Represents significance at (*p *≤ 0.007)

*CE *= Constant error, *TE *= Total error, *SEE *= Standard error of estimate, *r *= Pearson product-moment correlation coefficient, Limits = 95% limits of agreement (CE ± 1.96 SD of residual scores (predicted-actual))

HW = Hydrostatic weighing

BP = BOD POD^®^

NIR = Near-infrared interactance (Futrex 6100/XL)

BIA = Bioelectrical impedance analysis (RJL Systems Quantum II)

AK = Computer-generated from BodyGram™ Version 1.31 (Akern Bioresearch)

Lohman [41] - FFM (kg) = 0.476 (HT^2^/R) + 0.295 (BW) + 5.49

SF3JPW [45] - BD = 1.0994921 - 0.0009929 × X1 + 0.0000023 × X1^2 ^- 0.0001392 × age

SF3WB [22] - BD = 1.06234 - 0.00068 × subscapular - 0.00039 × triceps - 0.00025 × thigh

FFM = Fat-free mass

HT = Height (cm)

BW = Body weight (kg)

R = BIA resistance (Ω)

% Fat = (BW - FFM)/BW

X1 = Sum of skinfolds (triceps, suprailium, thigh)

BD = Body density

%fat [6] = (4.57/BD - 4.142) × 100.

All HW measurements were performed by an investigator who had previously demonstrated an intraclass correlation coefficient (ICC) of 0.99 with a standard error of measurement (*SEM*) < 1.0%fat. These values are comparable to those reported by other laboratories [15,20]. Previous test-retest measurements for HW produced a *SEM *of 0.74%fat and 0.002729 g/cc for BD.

### Air-displacement plethysmography (BOD POD^®^)

Before each test, the BP was calibrated according to the manufacturer's instructions with the chamber empty using a cylinder of known volume (49.558 L). The subject, wearing a tight fitting bathing suit and a swimming cap, entered and sat in the fiberglass chamber. The BP was sealed, and the subject breathed normally for 20 seconds while body volume was measured. Next, the subject was connected to a breathing tube internal to the system to measure thoracic gas volume. The subject resumed tidal breathing cycles; a valve in the circuit caused a momentary occlusion of the airway, during which the subject gently "puffed". This effort produced small pressure fluctuations in the airway and chamber that were used to determine thoracic gas volume. This value was used to correct body volume for thoracic gas volume. All BP measurements were performed by a BOD POD^® ^certified investigator who had previously demonstrated a *SEM *of 0.48%fat.

### Near-infrared interactance

The Futrex^® ^6100/XL was used to measure the %fat of each subject according to the procedures recommended by the manufacturer (Futrex^®^, Hagerstown, MD). This device emits infrared light of six specific wavelengths (810, 910, 932, 944, 976, and 1,023 nm) into the anterior midline of the *biceps brachii *midway between the *antecubital fossa *and *acromion *process of the right arm. A silicon-based detector then measured the intensity of the re-emitted light, which was expressed as optical density. Percent body fat was estimated using a pre-programmed generalized multiple regression equation that included height, weight, and optical density values. The specific equation used to calculate %fat was not available from the manufacturer. The instrument was calibrated prior to each measurement with the manufacturer-supplied optical standard. Previous test-retest measurements for the Futrex^® ^6100/XL produced a *SEM *of 0.74%fat.

### Bioelectrical impedance analysis

BIA analysis was performed using the Quantum II Bioelectrical Body Composition Analyzer following the procedures recommended by the manufacturer (RJL Systems, Clinton Twp, MI). Percent body fat was estimated from the resistance (Ω) and reactance (Ω) values produced by the Quantum II. After at least 1 to 2 minutes of supine rest, within 10 minutes, resistance and reactance measurements were taken while the subjects laid supine on a table with their arms ≥ 30 degrees away from their torso with their legs separated from each other. Electrodes were placed at the distal ends of the subject's right hand and foot following the manufacturer guidelines. Excess body hair was removed prior to electrode placement, and the site (skin) was cleaned with alcohol. The average of two trials within ± 5 Ω was used to represent the subjects resistance (Rz) and reactance (Xc) values. The average of resistance values and the subject's height, weight, sex, and age were entered into a computer program (BodyGram™ Version 1.31, Akern Bioresearch, Pontassieve (FL), Italy) to estimate %fat (BIA-AK). Additionally, fat-free mass was estimated from Lohman's prediction equation (BIA-Lohman) specific to this population and converted to %fat (Table 2). Previous test-retest measurements for BIA-AK produced a *SEM *of 0.68%fat.

### Skinfolds

Skinfold thickness measurements were taken on the right side of the body with a calibrated Lange caliper by an investigator who had previously demonstrated a test-retest reliability of *r *> 0.90. Measurements were taken according to the recommendations of Jackson and Pollock [21] at the sites for triceps, suprailium, subscapular, and thigh. Body density values were calculated using the generalized skinfold equation of Jackson et al. [21] and Wilmore and Behnke [22]. Percent body fat was calculated from BD using the revised formula of Brozek et al. [6] (Table 2).

### Bioimpedance spectroscopy

Total body water (TBW) was analyzed using Bioimpedance spectroscopy via the ImpediMed Imp™ SFB7 device following the procedures recommended by the manufacturer (ImpediMed, Queensland, Australia). BIS uses a range of frequencies that encompass both low and high ranges that allow electrical current to pass around and through each cell. This technique, explained elsewhere [23], has produced valid measurement of TBW when compared to a criterion method such as deuterium oxide [23]. After at least 1 to 2 minutes of supine rest, within 10 minutes, total body water estimates were taken while the subjects laid supine on a table following the procedures as described for the BIA. The average of two trials within ± 0.05 liters were used to represent the subject's TBW. Prior to analysis each subject's height, weight, age, and sex were entered into the SFB7 device. Internal to the device, the SFB7 utilized 256 frequencies and a complex impedance plot to estimate TBW. Previous test-retest measurements for the SFB7 BIS produced a *SEM *of 0.48 liters. Recently examined in our laboratory, the BIS device used in the current study was compared to deuterium oxide for estimating TBW in a heterogeneous sample of men and women (*n *= 30, 23.8 ± 4 years, 174.47 ± 7.34 cm, 73.4 ± 18.45 kg, 23.10 ± 5.77%fat, x¯
 MathType@MTEF@5@5@+=feaafiart1ev1aaatCvAUfKttLearuWrP9MDH5MBPbIqV92AaeXatLxBI9gBaebbnrfifHhDYfgasaacPC6xNi=xH8viVGI8Gi=hEeeu0xXdbba9frFj0xb9qqpG0dXdb9aspeI8k8fiI+fsY=rqGqVepae9pg0db9vqaiVgFr0xfr=xfr=xc9adbaqaaeGacaGaaiaabeqaaeqabiWaaaGcbaGafmiEaGNbaebaaaa@2D64@ ± SD). The results demonstrated a non-significant *CE *(-0.56 L, *p *> 0.05) and a high correlation (*r *= 0.97), which is similar to results obtained in other laboratories [23-25].

### 3C calculation

The following equation was used to calculate the criterion %fat via the 3C model [5] (BD from HW, TBW from BIS):

%fat = [(2.118/BD) - (0.78 × TBW/Body Weight (kg)) - 1.354] × 100

The total error of measurement (*TEM*) for the 3C model was calculated from the following equation [26]:

3C *TEM *= (TBW *SEM*^2 ^+ HW BD *SEM*^2^)^1/2^

3C *TEM *= (0.48^2 ^+ 0.002729^2^)^1/2^

3C *TEM *= 0.1152 %fat

### Data analysis

Validity of %fat estimates (BP, HW, NIR, BIA-AK, BIA-Lohman, SF3JPW, and SF3WB) was based on an evaluation of predicted values versus the criterion (actual value) 3C by calculating the constant error (*CE *= actual (3C) - predicted %fat), *r *value, standard error of estimate (SEE=SD1−r2)
 MathType@MTEF@5@5@+=feaafiart1ev1aaatCvAUfKttLearuWrP9MDH5MBPbIqV92AaeXatLxBI9gBaebbnrfifHhDYfgasaacPC6xNi=xH8viVGI8Gi=hEeeu0xXdbba9frFj0xb9qqpG0dXdb9aspeI8k8fiI+fsY=rqGqVepae9pg0db9vqaiVgFr0xfr=xfr=xc9adbaqaaeGacaGaaiaabeqaaeqabiWaaaGcbaGaeiikaGIaem4uamLaemyrauKaemyrauKaeyypa0Jaee4uamLaeeiraq0aaOaaaeaacqaIXaqmcqGHsislcqqGYbGCdaahaaWcbeqaaiabikdaYaaaaeqaaOGaeiykaKcaaa@389D@, total error (TE=∑[predicted−actual]2/n)
 MathType@MTEF@5@5@+=feaafiart1ev1aaatCvAUfKttLearuWrP9MDH5MBPbIqV92AaeXatLxBI9gBaebbnrfifHhDYfgasaacPC6xNi=xH8viVGI8Gi=hEeeu0xXdbba9frFj0xb9qqpG0dXdb9aspeI8k8fiI+fsY=rqGqVepae9pg0db9vqaiVgFr0xfr=xfr=xc9adbaqaaeGacaGaaiaabeqaaeqabiWaaaGcbaGaeiikaGIaemivaqLaemyrauKaeyypa0ZaaOaaaeaadaaeabqaaiabcUfaBjabbchaWjabbkhaYjabbwgaLjabbsgaKjabbMgaPjabbogaJjabbsha0jabbwgaLjabbsgaKjabgkHiTiabbggaHjabbogaJjabbsha0jabbwha1jabbggaHjabbYgaSjabc2faDnaaCaaaleqabaGaeGOmaidaaOGaei4la8IaeeOBa4galeqabeqdcqGHris5aaWcbeaakiabcMcaPaaa@4E2C@, and the similarity between the standard deviation (SD) of predicted and actual values [9,27]. The mean difference (*CE*) between the predicted (BP, HW, NIR, BIA-AK, BIA-Lohman, SF3JPW and SF3WB) and actual (3C) %fat values was analyzed using dependent *t*-tests with the Bonferroni alpha adjustment (*p *≤ 0.006) [28]. Additionally, the method of Bland and Altman [29] was used to identify the 95% limits of agreement between the criterion and predicted %fat values.

## Results

The 3C model was considered the criterion measure; the average %fat determined by the 3C was 26.3 ± 4.6 %fat. Presented in Table 2 are the results of the validation analyses. Constant error values ranged from 2.6 (SF3JPW) to -0.1 (BIA-AK) with significant *CE *differences (*p *≤ 0.006) detected for HW and SF3JPW. The lowest validity coefficient was 0.80 (SF3JPW) and the highest was 0.94 (HW), while the *SEE *values ranged from 1.6%fat (HW) to 2.8%fat (SF3JP3). All laboratory and field methods resulted in acceptable *TE *values (≤ 3.8%fat). Of the field methods, the 95% limits of agreement were the largest for both skinfold equations and NIR (≥ 6.0 to -2.8%fat) while the BIA-AK and BIA-Lohman produced smaller limits of agreement (≤ 4.7 to -3.7%fat).

## Discussion

All methods used to estimate body composition in this study produced acceptable *TE *values and are acceptable body composition techniques for use in college-age Caucasian women. The results of the current study suggest that the BP and HW are valid laboratory methods when compared to the 3C model to estimate %fat in this population. In addition, all field methods produced acceptable *TE *and *SEE *values ranging from 2.1 – 3.8 %fat for *TE *and 1.6 – 2.8 %fat for the *SEE*. As a result, all were considered valid methods for estimating %fat in this population. However, the large limits of agreement suggest that caution should be used when relying on any one of these methods as a technique to identify %fat in small groups or individuals.

### Laboratory methods

#### Hydrostatic weighing

Contrary to the findings of Clasey et al. [8], who compared HW to a 4C model (*TE *= 5.17 %fat) in young women, HW produced acceptable *TE *values (*TE *= 2.4%). However, in agreement with Clasey et al. [8], HW produced a similar *r *value (*r *= 0.87) and produced a significant *CE *(*CE *= -3.4 %fat) compared to the current findings (*r *= 0.94, *CE *= 1.5 %fat). Nonetheless, Clasey et al. [8] concluded that HW overestimated %fat compared to a 4C model, while the current investigation found HW to underestimate %fat compared to the 3C. The discrepancies of these findings could be a result of the models used. Clasey et al. [8] used the 4C model, which utilizes bone mineral content, as well as TBW and BD. A similar agreement was found between the investigation of Fields et al. [30] (*TE *= 2.4%fat) in adult women (19–54 years) and the current investigation (*TE *= 2.4%fat). Moreover, Fields et al. [30] demonstrated a similar *r *(*r *= 0.92) value compared to the current investigation (*r *= 0.91). However, BD measured via HW was used in the criterion 3C equation, inflating the correlation between methods by increasing the *r *values. Nonetheless, several studies have compared HW to multi-compartment models in which HW measurements were included [31-33]. Ultimately, it appears from the current investigation that HW is a valid method for estimating %fat in this population. However, the 95% limits of agreement suggest that HW may over-predict %fat by as much as 2.3%fat and under-predict by as much as 5.3%fat. These individual variations are most likely due to the use of a 2C model which does not require an estimate of TBW. Therefore, caution should be used when relying on HW as a method to identify %fat in small groups or individuals.

#### BOD POD^®^

Due to the ease in procedure, speed, and improved subject compliance, BP provides an attractive alternative to HW. Additionally, research has shown that participants prefer the BP over HW [34]. Results of this study demonstrated high validity coefficient (*r *= 0.91) and "excellent" *SEE *(1.9%fat) and *TE *(2.3%fat) values [9]. Although the BP is a relatively new device, a number of studies have examined the validity when compared to HW in females with contradictory findings [30,35-37]. However, only limited research is available on the validity of the BP in college-age women compared to multiple-compartment models, specifically the 3C model. Nonetheless, there is agreement with the work of Fields et al. [30] (*TE *= 2.3%fat) in adult women (19–54 years) and the current investigation (*TE *= 2.3%fat), which suggests that the BP is an acceptable laboratory method for estimating %fat in college women. The current findings suggest, as with HW, that BP is a valid method for estimating %fat in college-age women. However, the 95% limits of agreement suggest that BP may over-predict %fat by as much as 4.8%fat and under-predict by as much as 4.1%fat. These individual variations are most likely due to the use of a 2C model which does not require an estimate of TBW. Therefore, caution should be used when relying on BP as a method to identify %fat in small groups or individuals.

### Field methods

#### Near-infrared interactance

The second aim of this study was to examine the validity of the newly-developed NIR (Futrex^®^6100/XL) employing six wavelengths to estimate %fat. In 1984, Conway et al. [10] measured infrared wavelengths from 700 to 1100 nm and determined that the peak absorption of pure fat occurred at 930 nm and pure water at 970 nm. Based on this research, the Futrex^®^5000 utilized infrared wavelengths at 940 and 950 nm to measure optical density. The updated model of the Futrex^® ^(6100/XL) NIR device used in the current investigation employs six different wavelengths at 810, 910, 932, 944, 976, and 1023 nm to estimate %fat [38]. The Futrex^®^6100/XL has advantages over previous models because the wavelengths encompass the same range as in the original research by Conway et al. [10] (700–1100 nm). Results from the current study indicated no difference between NIR and the 3C model for %fat estimations and produced acceptable *SEE *(2.7%fat) and *TE *(2.7%fat) values, as well as a good validity coefficient (*r *= 0.82). Though other studies have validated earlier models (Futrex^®^5000 and Futrex^®^1000) in females compared to HW [11,12,14,15]; to our knowledge this is the first complete study to compare the new NIR device to any criterion method. However, previous studies concluded that neither the Futrex^®^1000 or Futrex^®^5000 were acceptable methods for estimating %fat with reported *TE *values > 4%fat [11,12,14]. Stout et al. [15] was the only investigation that found acceptable agreement between the Futrex^®^5000 and HW in women 17 to 29 years of age (20.1 ± 2.3 years). Nonetheless, Stout et al. [15] concluded that, even though the *TE *value of 3.9%fat is considered acceptable for the gross estimation of body fat, the Futrex^®^5000 substantially condensed the distribution of body fat and constantly overestimated %fat. In agreement with Stout et al. [15], the current investigation produced an acceptable *TE *value (*TE *= 2.7%fat). However, contrary to the conclusions of Stout et al. [15] and the Futrex^®^5000, the Futrex^®^6100/XL appears to be a favorable device for measuring %fat in college women and can be considered a valid field method for estimating %fat in this population. Additionally, the validity coefficient (*r *= 0.82) using the Futrex^®^6100/XL is the highest reported of any prior research or Futrex^® ^model (*r *< 0.63) [11,12,14,15].). It appears that the six wavelengths utilized in the Futrex^®^6100/XL compared to the two wavelengths for the Futrex^®^5000 and Futrex^®^1000 improved the accuracy for estimating %fat in this population. Based on the current findings, the Futrex^®^6100/XL appears to be a valid method for estimating %fat in college-age women; however, more research is required to validate our results. Additionally, the 95% limits of agreement suggest that NIR may over-predict %fat by as much as 4.2%fat and under-predict by as much as 6.2%fat. These individual variations are most likely due to the use of a single site measurement. Therefore, caution should be used when relying on NIR as a method to identify %fat in small groups or individuals. Future research should examine the validity of the Futrex^®^6100/XL in different populations that include various races, sex, ages, degrees of fatness, and include multiple measurements sites.

#### Bioelectrical impedance

Results of previous studies on BIA validity for estimating %fat in college women have been equivocal [11,12,15-18]. Single frequency (50-kHz) BIA devices, such as the RJL Quantum II, are based on the work of Thomasett [39], who implemented the use of a low-level electrical current and measured the opposition of flow. This opposition of electrical current through the body is directly related to its composition of water, fat, and lean tissue. Since the electrolytes in water are good conductors of electric current, the opposition of electrical flow can be used to estimate TBW and lean body mass, with the assumption that lean body mass has ≈ 73% water [40]. Total body weight and lean body mass are used in the calculation of %fat; however, TBW differences may exist across race, sex, age, and health status. Therefore, body composition experts have suggested using BIA population-specific equations to estimate %fat [27]. Lohman [41] developed a population-specific equation for women 18–30 years of age that includes BIA measurement of resistance, height, and weight (BIA-Lohman). A more technical method to assess body composition from BIA uses vectors as described by Piccoli et al. [42] and phase angles as described by Barbosa-Silva and Barros [43], which require BIA-measured resistance and reactance values. The BodyGram™ (Version 1.31, Akern Bioresearch) computer program utilizes resistance and reactance values to estimate %fat (BIA-AK) via vectors and phase angles. BIA-Lohman and BIA-AK equations were developed to estimate %fat in women 18–30 years of age and, therefore, should produce similar %fat values to the 3C model. However, to the best of our knowledge, no research has validated BIA-AK %fat estimations in healthy college-age Caucasian women.

The *SEE *(1.9 %fat) values reported in this study for BIA-AK (RJL Quantum II, RJL Systems) were substantially lower than those reported by Lukaski et al. [17] (*SEE *= 3.1%fat) (Model BIA-101, RJL Systems) and Stout et al. [15] (*SEE *= 3.5%fat) (Model BIA-106, RJL Systems). Additionally, the current *r *value (*r *= 0.92) was greater than that of both investigations by Stout et al. [15] and Lukaski et al. [17] (*r *= 0.88). However, unacceptable values (*SEE *> 4.0 %fat) have been reported by Eaton et al. [11] and Jackson et al. [16] (BIA106, RJL Systems and BIA103B, RJL Systems, respectively). Regarding *TE*, current results (1.8 %fat) are the lowest reported, though Heyward et al. [12] reported acceptable values (3.8 %fat), while Jackson et al. [16] and Stout et al. [15] both reported unacceptable values > 6.4% fat. These discrepancies were most likely due to the variation of devices used and the subsequent equations or methods used to estimate %fat. It appears that the current method used to estimate %fat (BodyGram™ Version 1.31, Akern Bioresearch) is an acceptable procedure to estimate the %fat of college-age women.

The BIA-Lohman population-specific equation produced similar results to the BIA-AK. Past research has produced conflicting results when the BIA-Lohman equation was compared to HW. Stout et al. [15] (women 20.1 ± 2.3 years) found the BIA-Lohman equation to be unacceptable with a *TE *of 5.3%fat, while Heyward et al. [12] found an acceptable *TE *of 3.78% fat in non-obese women (20–72 years < 30% fat). In agreement with Heyward et al. [12], the current investigation found an acceptable *TE *(*TE *= 2.1%) value. Additionally, compared to both studies (*r *< 0.64), the current *r *value is much higher (*r *= 0.93).

To the best of our knowledge, there has been no evaluation of the validity of any BIA method of predicting %fat in college women compared to a multiple-compartment model. Therefore, this is the first investigation showing the strong agreement between the 3C model or any multiple-component model and both BIA-AK and BIA-Lohman in this population. The lower *TE *values in the current investigation compared to past literature comparing BIA to HW are most likely due to the utilization of TBW in the criterion 3C model. The relationship between fat free mass (FFM) and TBW has been well established [40]. The strong correlation between resistance and reactance measured by BIA and FFM measured by BIA could have improved both BIA values in contrast to past literature in which TBW was not estimated. However, in the current investigation, both BIA-AK and BIA-Lohman produced acceptable *TE *values compared to HW (*TE *= 3.33%fat), suggesting that TBW may increase the accuracy of BIA procedures. Ultimately, the removal of TBW in the criterion method did not produce unacceptable BIA *TE *values (*TE *< 4.0%fat). Future research is needed to re-evaluate the validity of previously-used equations and BIA devices compared to a multiple-compartment models that utilize TBW. Furthermore, it appears that both BIA-AK and BIA-Lohman are acceptable field methods for estimating %fat in college women. However, the 95% limits of agreement suggest that both BIA-AK and BIA-Lohman may over-predict %fat by as much as 3.7 and 2.8%fat and under-predict by as much as 3.5 and 4.7%fat, respectively. These individual variations are most likely attributed to deviations in intra-cellular fluid which a single frequency (50-kHz) BIA device cannot detect. Therefore, caution should be used when relying on BIA as a method to identify %fat in small groups or individuals.

#### Skinfolds

This is the first investigation of which we are aware that has compared either the SF3JPW or SF3WB to the 3C model in college women. Previous research has compared the SF3JPW skinfold prediction equations to HW with similar results in women [12,15,44,45]. In agreement with these investigations (*TE *= 2.4–3.85%fat, *r *= 0.65–0.88, *SEE *= 2.1–3.6%fat), the current findings were acceptable (SF3JPW *TE *= 3.8%fat, *r *= 0.80, *SEE *= 2.8%fat), concluding that the SF3JPW skinfold quadratic equation is not only valid compared to HW but also compared to the 3C model, which includes TBW. Therefore, the SF3JPW is an acceptable field method for predicting %fat in college women. However, an investigation by Brandon [46] found similar *r *(*r *= 0.82) and *SEE *(*SEE *= 2.6) values with an unacceptable *TE *(*TE *= 4.9%fat).

The SF3WB equation has not been investigated to the same extent as the SF3JPW equation. An investigation by Brandon [46] found an acceptable *TE *compared to HW in women (21 ± 2.9) (*TE *= 3.8%fat) and similar *r *(*r *= 0.82) and *SEE *(*SEE *= 2.6%fat) values with the current investigation (*r *= 0.88, *SEE *= 2.2%fat, *TE *= 2.8%fat). The linear equation of Wilmore and Behnke [22] appears to be an acceptable method for predicting %fat in college women. Both the quadratic equation of Jackson et al. [45] and linear equation of Wilmore and Behnke [22] are acceptable for estimating %fat in this population and, due to the low cost and ease of use, may be a more attractive field method compared to more expensive field devices such as NIR and BIA. However, the 95% limits of agreement suggest that both SF3JP and SFWB may over-predict %fat by as much as 2.8 and 4.5%fat and under-predict by as much as 8.1 and 6.0%fat, respectively. These individual variations are most likely attributed to deviations in subcutaneous fat distribution. Therefore, caution should be used when relying on SF equations as a method to identify %fat in small groups or individuals.

## Conclusion

Both two-compartment laboratory methods (BP, HW) produced acceptable *TE *values when compared to the 3C model of Siri [5]. However, due to the large limits of agreement, the use of 2C models may not be appropriate when attempting to identify %fat in small groups or individuals due to the individual variations of TBW [4,8,33]. Wang et al. [4] determined that the 3C model used in the current investigation along with 4C models were some of the best methods to use as criteria %fat estimates compared to the 6C model and concluded that these 2C models (BP, HW) may not be appropriate across all age, sex, and disease groups for estimating %fat. However, the current investigation provides data that validate the use of these 2C models (BP, HW) for use in college-age women. However, caution should be used when utilizing one of these 2C models in a research setting when %fat or other body composition compartments (fat-free mass, TBW) are being estimated for small groups or individual college-age Caucasian women.

Limitations of the current study include the estimates of %fat from the 3C model. Since %fat values from the 3C model included measurements from HW, there could potentially be greater agreement between these methods and the 3C model. Additionally, multiple-compartment models, with greater complexity than the 3C, such as the 4, 5 and 6C model are better criterion methods, and, thus, should be used to validate the methods included in the current study in this population. Furthermore, TBW measurements were obtained via BIS which is a valid measure but not a criterion method. The possibility exists that the use of a criterion method, such as deuterium oxide, to measure TBW could influence the values found in the current investigation. Nonetheless, there are inherent errors associated with all body composition measurements, as the "true" value of body fat is unmeasurable [4]. The use of multiple compartment models as criterion methods reduce the errors associated with 2C models, and single devices such as dual-energy X-ray absorptiometry by accounting for total body water, which is the largest molecular level component of the body [4]. Furthermore, these multiple compartment models that include TBW measurements (Siri 3C, 4C, and 5C models) "form a core with the 6C model" and are not known to be age, sex, race, and health status dependent [4].

In conclusion, this study provides original data regarding the validity of laboratory and field methods. Our data suggest BP is an acceptable laboratory method to use when HW or multiple-compartment models are not available or subject compliance is a potential problem. Furthermore, our results indicate that NIR, BIA-AK, BIA-Lohman, SF3JPW, and SF3WB are valid alternatives when laboratory methods are unavailable for estimating %fat in college-age Caucasian women.

## Competing interests

The author(s) declare that they have no competing interests.

## Authors' contributions

JM, HH, ST, JC and JS participated in the study design and helped draft the manuscript while aiding in data collection. MT, MK, MR, ER, SJK, VD, AW, AS participated in data collection and analysis. Additionally, all authors read and approved the final manuscript.

## References

[B1] Durnin JV, Womersley J (1974). Body fat assessed from total body density and its estimation from skinfold thickness: measurements on 481 men and women aged from 16 to 72 years. The British journal of nutrition.

[B2] Burke LM, Loucks AB, Broad N (2006). Energy and carbohydrate for training and recovery. Journal of sports sciences.

[B3] Nelson KM, Weinsier RL, Long CL, Schutz Y (1992). Prediction of resting energy expenditure from fat-free mass and fat mass. Am J Clin Nutr.

[B4] Wang ZM, Deurenberg P, Guo SS, Pietrobelli A, Wang J, Pierson RN, Heymsfield SB (1998). Six-compartment body composition model: inter-method comparisons of total body fat measurement. Int J Obes Relat Metab Disord.

[B5] Siri WE, Brozek J, Henschel A (1961). Body composition from fluid spaces and density. Analysis of methods.. Techniques for Measuring Body Composition.

[B6] Brozek J, Grande F, Anderson JT, Keys A (1963). Densitometric Analysis of Body Composition: Revision of Some Quantitative Assumptions. Ann N Y Acad Sci.

[B7] Fuller NJ, Jebb SA, Laskey MA, Coward WA, Elia M (1992). Four-component model for the assessment of body composition in humans: comparison with alternative methods, and evaluation of the density and hydration of fat-free mass. Clin Sci (Lond).

[B8] Clasey JL, Kanaley JA, Wideman L, Heymsfield SB, Teates CD, Gutgesell ME, Thorner MO, Hartman ML, Weltman A (1999). Validity of methods of body composition assessment in young and older men and women. J Appl Physiol.

[B9] Heyward VH, Wagner DR (2004). Applied Body Composition Assessments.

[B10] Conway JM, Norris KH, Bodwell CE (1984). A new approach for the estimation of body composition: infrared interactance. Am J Clin Nutr.

[B11] Eaton AW, Israel RG, O'Brien KF, Hortobagyi T, McCammon MR (1993). Comparison of four methods to assess body composition in women. Eur J Clin Nutr.

[B12] Heyward VH, Cook KL, Hicks VL, Jenkins KA, Quatrochi JA, Wilson WL (1992). Predictive accuracy of three field methods for estimating relative body fatness of nonobese and obese women. International journal of sport nutrition.

[B13] Israel RG, Houmard JA, O'Brien KF, McCammon MR, Zamora BS, Eaton AW (1989). Validity of a near-infrared spectrophotometry device for estimating human body composition. Res Q Exerc Sport.

[B14] McLean KP, Skinner JS (1992). Validity of Futrex-5000 for body composition determination. Medicine and science in sports and exercise.

[B15] Stout JR, Housh TJ, Eckerson JM, Johnson GO, Betts NM (1996). Validity of methods for estimating percent body fat in young women. Strength and Conditioning Research.

[B16] Jackson AS, Pollock ML, Graves JE, Mahar MT (1988). Reliability and validity of bioelectrical impedance in determining body composition. J Appl Physiol.

[B17] Lukaski HC, Bolonchuk WW, Hall CB, Siders WA (1986). Validation of tetrapolar bioelectrical impedance method to assess human body composition. J Appl Physiol.

[B18] Segal KR, Van Loan M, Fitzgerald PI, Hodgdon JA, Van Itallie TB (1988). Lean body mass estimation by bioelectrical impedance analysis: a four-site cross-validation study. Am J Clin Nutr.

[B19] Wilmore JH (1969). A simplified method for determination of residual lung volumes. J Appl Physiol.

[B20] Thomas TR, Cook PL (1978). A simple inexpensive method for estimating underwater weight. British journal of sports medicine.

[B21] Jackson AS, Pollock ML (1985). Practical assessment of body composition. Phys Sport Med.

[B22] Wilmore JH, Behnke AR (1970). An anthropometric estimation of body density and lean body weight in young women. Am J Clin Nutr.

[B23] Matthie J, Zarowitz B, De Lorenzo A, Andreoli A, Katzarski K, Pan G, Withers P (1998). Analytic assessment of the various bioimpedance methods used to estimate body water. J Appl Physiol.

[B24] Sardinha LB (2003). Usefulness of age-adjusted equations to estimate body fat with air displacement Plethysmography in male adolescent athletes. Acta Diabetol.

[B25] Van Loan MD, Withers P, Matthie J, Mayclin PL (1993). Use of bioimpedance spectroscopy to determine extracellular fluid, intracellular fluid, total body water, and fat-free mass. Basic Life Sciences.

[B26] Heymesfield SB, Lohman TG, Wang Z, Going SB (2005). Human body composition.

[B27] Lohman TG (1996). Human Body Composition.

[B28] Keppel GTDW (2004). Design and Analysis: A Researchers Handbook.

[B29] Bland JM, Altman DG (1986). Statistical methods for assessing agreement between two methods of clinical measurement. Lancet.

[B30] Fields DA, Wilson GD, Gladden LB, Hunter GR, Pascoe DD, Goran MI (2001). Comparison of the BOD POD with the four-compartment model in adult females. Medicine and science in sports and exercise.

[B31] Arngrimsson S, Evans EM, Saunders MJ, Ogburn CL, Lewis RD, Cureton KJ (2000). Validation of body composition estimates in male and female distance runners using estimates from a four-component model. Am J Hum Biol.

[B32] Prior BM, Modlesky CM, Evans EM, Sloniger MA, Saunders MJ, Lewis RD, Cureton KJ (2001). Muscularity and the density of the fat-free mass in athletes. J Appl Physiol.

[B33] Withers RT, LaForgia J, Pillans RK, Shipp NJ, Chatterton BE, Schultz CG, Leaney F (1998). Comparisons of two-, three-, and four-compartment models of body composition analysis in men and women. J Appl Physiol.

[B34] Demerath EW, Guo SS, Chumlea WC, Towne B, Roche AF, Siervogel RM (2002). Comparison of percent body fat estimates using air displacement plethysmography and hydrodensitometry in adults and children. Int J Obes Relat Metab Disord.

[B35] Biaggi RR, Vollman MW, Nies MA, Brener CE, Flakoll PJ, Levenhagen DK, Sun M, Karabulut Z, Chen KY (1999). Comparison of air-displacement plethysmography with hydrostatic weighing and bioelectrical impedance analysis for the assessment of body composition in healthy adults. Am J Clin Nutr.

[B36] Levenhagen DK, Borel MJ, Welch DC, Piasecki JH, Piasecki DP, Chen KY, Flakoll PJ (1999). A comparison of air displacement plethysmography with three other techniques to determine body fat in healthy adults. Jpen.

[B37] McCrory MA, Gomez TD, Bernauer EM, Mole PA (1995). Evaluation of a new air displacement plethysmograph for measuring human body composition. Medicine and science in sports and exercise.

[B38] Futrex I (2007). Quality Systems Regulations: Futrex's internal Manual..

[B39] Thomasett A (1962). Bio-electrical properties of tissue impedance measurements. Lyon Medical.

[B40] Wang Z, Deurenberg P, Wang W, Pietrobelli A, Baumgartner RN, Heymsfield SB (1999). Hydration of fat-free body mass: review and critique of a classic body-composition constant. Am J Clin Nutr.

[B41] Lohman TG (1992). Advances in Body Composition Assessment..

[B42] Piccoli A, Nigrelli S, Caberlotto A, Bottazzo S, Rossi B, Pillon L, Maggiore Q (1995). Bivariate normal values of the bioelectrical impedance vector in adult and elderly populations.. Am J Clin Nutr.

[B43] Barbosa-Silva MCG, Barros AJD (2005). Bioelectrical impedance analysis in clinical practice: a new perspective on its use beyond body composition equations. Curr Opin Clin Nutr Metab.

[B44] Eckerson JM, Stout JR, Evetovich TK, Housh TJ, Johnson GO, Worrell N (1998). Validity of Self-Assessment Tequniques for Estimating Percent Fat in Men and Women. J Strength and Cond Res.

[B45] Jackson AS, Pollock ML, Ward A (1980). Generalized equations for predicting body density of women. Medicine and science in sports and exercise.

[B46] Brandon LJ (1998). Comparison of existing skinfold equations for estimating body fat in African American and white women. Am J Clin Nutr.

